# Longitudinal association between astrocyte function and glucose metabolism in autosomal dominant Alzheimer’s disease

**DOI:** 10.1007/s00259-018-4217-7

**Published:** 2018-12-04

**Authors:** Stephen F. Carter, Konstantinos Chiotis, Agneta Nordberg, Elena Rodriguez-Vieitez

**Affiliations:** 1 Wolfson Molecular Imaging Centre, Division of Neuroscience and Experimental Psychology, University of Manchester, Manchester, M20 3LJ UK; 2 Department of Neurobiology, Care Sciences and Society, Division of Clinical Geriatrics, Karolinska Institutet, 141 52 Stockholm, Sweden; 3 Theme Neurology, Karolinska University Hospital, 171 76 Stockholm, Sweden; 4 Theme Aging, Karolinska University Hospital, 141 86 Stockholm, Sweden

**Keywords:** Astrocytosis, Autosomal dominant Alzheimer’s disease, ^11^C-Deuterium-l-deprenyl, ^18^F-Fluorodeoxyglucose, Monoamine oxidase B, PET

## Abstract

**Purpose:**

The spatial resolution of ^18^F-fluorodeoxyglucose PET does not allow the specific cellular origin of its signal to be determined, but it is commonly accepted that transport and trapping of ^18^F-fluorodeoxyglucose reflects neuronal glucose metabolism. The main frameworks for the diagnosis of Alzheimer’s disease suggest that hypometabolism measured with ^18^F-fluorodeoxyglucose PET is a biomarker of neuronal injury and neurodegeneration. There is preclinical evidence to suggest that astrocytes contribute, at least partially, to the in vivo ^18^F-fluorodeoxyglucose PET signal. However, due to a paucity of PET tracers for imaging astrocytic processes, the relationship between astrocyte function and glucose metabolism in human brain is not fully understood. The aim of this study was to investigate the longitudinal association between astrocyte function and glucose metabolism in Alzheimer’s disease.

**Methods:**

The current investigation combined longitudinal PET data from patients with autosomal dominant Alzheimer’s disease, including data on astrocyte function (^11^C-deuterium-l-deprenyl binding) and glucose metabolism (^18^F-fluorodeoxyglucose uptake). Research participants included 7 presymptomatic and 4 symptomatic mutation carriers (age 44.9 ± 9.8 years and 58.0 ± 3.7 years, respectively) and 16 noncarriers (age 51.1 ± 14.2 years). Eight carriers and eight noncarriers underwent longitudinal follow-up PET imaging at an average of 2.8 ± 0.2 and 3.0 ± 0.5 years from baseline, respectively.

**Results:**

Longitudinal decline in astrocyte function as measured using ^11^C-deuterium-l-deprenyl PET was significantly associated with progressive hypometabolism (^18^F-fluorodeoxyglucose uptake) in mutation carriers; no significant association was observed in noncarriers.

**Conclusion:**

The emerging data shift the accepted wisdom that decreases in cerebral metabolism measured with ^18^F-fluorodeoxyglucose solely reflect neuronal injury, and places astrocytes more centrally in the development of Alzheimer’s disease.

**Electronic supplementary material:**

The online version of this article (10.1007/s00259-018-4217-7) contains supplementary material, which is available to authorized users.

## Introduction

The seminal study of Sokoloff et al. published in 1977 demonstrated that glucose metabolism can be measured in vivo in the rat brain using 2-deoxy-[^14^C]glucose [1]. Since then thousands of studies of the use of PET with the glucose analogue ^18^F-2-fluoro-2-deoxy-d-glucose (^18^F-FDG) have been published. ^18^F-FDG PET measures the rate at which the tracer is transported and trapped in cells, and cerebral glucose metabolic rates can be inferred by applying pharmacokinetic methods. The spatial resolution of PET is insufficient to allow the exact cellular origin of the signal to be determined, but it has been accepted that the trapping of ^18^F-FDG reflects neuronal metabolism. Based on this assumption ^18^F-FDG PET has gained wide acceptance for use in psychiatric and neurological clinical practice, where it is used to help identify the presence and progression of different brain diseases.

Alzheimer’s disease (AD) is a neurodegenerative disease whose clinical management includes the use of ^18^F-FDG PET. As the disease progresses, a characteristic topographical pattern of cerebral hypometabolism develops in the temporoparietal and posterior cingulate cortices. Recent diagnostic frameworks suggest that ^18^F-FDG hypometabolism is a biomarker of neuronal injury/neurodegeneration [2]. However, recent research challenges the long-held view that ^18^F-FDG PET solely reveals neuronal integrity.

Zimmer et al. [3] found compelling evidence that astrocytes contribute at least partially to the ^18^F-FDG PET signal measured in healthy rat brain, challenging the long-held neurocentric understanding of ^18^F-FDG metabolism. Zimmer et al. tested the hypothesis that astrocytes play a crucial role in coupling neuronal activity to glucose utilisation [4], a hypothesis based on in vitro findings in cultured mouse brain astrocytes [5]. The astrocyte–neuron lactate shuttle hypothesis [6] suggests that energy demands in neurons are met by lactate, which is produced in astrocytes and shuttled to neurons. There is ongoing debate about the veracity of this hypothesis [7, 8]. If proven to be valid, the hypothesis has significant implications for the interpretation of ^18^F-FDG PET brain imaging in a range of neurodegenerative diseases, but the authors indicate that there is as yet no in vivo human PET data to support their findings. This obviously provides the motivation to perform in vivo ^18^F-FDG PET imaging studies in humans to elucidate the cellular basis of ^18^F-FDG metabolism.

Few PET studies in humans have investigated the relationship between astrocytic processes and ^18^F-FDG PET findings, primarily because of a paucity of specific in vivo astrocyte biomarkers. A series of multitracer PET studies have measured glucose metabolism and aerobic glycolysis with ^18^F-FDG and ^15^O-labelled water, carbon monoxide and oxygen PET scans [9, 10]. Aerobic glycolysis, that is known to occur mostly in astrocytes and to play a role in biosynthesis and neuroprotection, has been observed to decline as tau accumulates in amyloid-positive cognitively normal individuals [9], suggesting that astrocyte dysfunction may contribute to tau-related neurodegeneration in preclinical AD. Similarly, reduced striatal aerobic glycolysis and low ^18^F-FDG uptake have been observed in patients with Huntington’s disease, suggesting that astrocyte dysfunction may contribute to hypometabolism in this disease [11].

In the absence of specific astrocyte PET biomarkers, monoamine oxidase B (MAO-B) has been used as a surrogate target. MAO-B is an enzyme that catalyses the oxidative deamination of monoamines, being overexpressed predominantly in astrocytes [12]. In vitro MAO-B concentration is correlated with various astrocyte markers in several neurodegenerative diseases [13], and has therefore been adopted as a marker of astrocyte function. In vivo MAO-B can be measured using ^11^C-deuterium-l-deprenyl (^11^C-DED) [14]. A cross-sectional PET study in humans using ^11^C-DED, ^18^F-FDG and ^11^C-Pittsburgh compound-B (^11^C-PiB) [15] demonstrated elevated ^11^C-DED binding in presymptomatic autosomal dominant AD (ADAD) mutation carriers; metabolism measured with ^18^F-FDG PET was largely preserved. In ADAD mutation carriers who were closer to the expected age of disease onset, both ^11^C-DED binding and ^18^F-FDG uptake were decreased relative to early presymptomatic levels. A longitudinal follow-up of the same ADAD cohort [16] revealed that both ^11^C-DED binding and ^18^F-FDG uptake declined longitudinally as the disease progressed. In contrast, ^11^C-PiB retention showed a diverging trajectory whereby fibrillary amyloid-β deposition increased with disease severity [16]. The results of regional linear mixed-effects models (LMEM) demonstrated significant rates of decline for both ^11^C-DED and ^18^F-FDG in mutation carriers, although these measures were not compared directly.

The studies by Schöll et al. [15] and Rodriguez-Vieitez et al. [16] suggest tantalisingly that in ADAD mutation carriers astrocyte function and metabolism are coupled as ADAD progresses. These studies provide tentative evidence that the in vivo ^18^F-FDG PET signal shares some common variance with astrocyte function, and that hypometabolism measured with PET could partly represent decreased glucose uptake into astrocytes. Neither of the studies [15, 16] was designed to test this hypothesis. The purpose of the study reported here was to test whether decreased ^18^F-FDG uptake is significantly associated with decreased ^11^C-DED binding (a marker of astrocyte function) in ADAD patients followed longitudinally.

## Materials and methods

### Participants

Participants from families carrying ADAD mutations were recruited as previously reported [15, 16]. The research subjects were part of a prospective longitudinal study on familial ADAD that has been ongoing at the Karolinska Institutet since 1993, in which subjects undergo repeated examinations over time, including clinical evaluation, a comprehensive neuropsychological assessment, neuroimaging, electroencephalography, apolipoprotein E (*APOE*) genotyping from blood samples, and collection of cerebrospinal fluid samples.

We have now conducted additional analyses to investigate the longitudinal relationship between MAO-B expression and glucose metabolism. The estimated years to symptom onset (EYO) was calculated for both mutation carriers and noncarriers by subtracting each subject’s age from the family-specific average age of onset [16]. In the study presented here, we analysed longitudinal PET data in 11 mutation carriers: seven presymptomatic (age 44.9 ± 9.8 years, EYO −10.1 ± 9.1 years) and four symptomatic (age 58.0 ± 3.7 years, EYO 0.8 ± 3.3 years). Eight carriers underwent follow-up PET imaging at 2.8 ± 0.2 years from baseline. Also, 16 noncarriers underwent baseline PET imaging (age 51.1 ± 14.2 years, EYO −4.4 ± 12.6 years), of whom eight underwent follow-up PET imaging at 3.0 ± 0.5 years. Symptomatic mutation carriers were diagnosed clinically as having either prodromal AD [2, 17] or AD dementia [18]. The presymptomatic mutation carriers had no cognitive dysfunction and did not fulfil the criteria for prodromal AD or AD dementia.

### MRI and PET image acquisition, processing and quantification

The ^11^C-DED PET tracer was produced and the PET and MRI image acquisition methods were as previously described [16, 19]. Briefly, dynamic ^11^C-DED and ^18^F-FDG PET images were acquired over 60 min and 45 min, respectively, on an ECAT EXACT HR+ PET/CT scanner (Siemens/CTI) and a GE Discovery ST PET/CT scanner following previously described procedures for radiotracer administration, PET image acquisition, reconstruction and motion correction. In each subject, a structural T1 MRI image was acquired within 3.8 ± 3.7 months of the ^11^C-DED PET scan. ^18^F-FDG scans were acquired on the same day as the ^11^C-DED PET scan, except in three subjects in whom the time between the two PET scans was less than 3 months. In each subject, the T1 MRI image was acquired using a 3-T Siemens Trio scanner and coregistered with the subject’s ^11^C-DED late-sum image (10–60 min) in native ^11^C-DED space using SPM8. Each subject’s ^18^F-FDG late-sum image (30–45 min) was coregistered with the T1 MRI image (which had been previously coregistered with native ^11^C-DED space). This T1 MRI image was segmented and a binary grey matter mask was created from the resultant probabilistic grey matter map (threshold 0.5). Using the inverse nonlinear transformation from this segmentation, a simplified probabilistic atlas [20] consisting of 12 bilateral regions of interest was registered from the Montreal Neurological Institute (MNI) space back into the subject’s native ^11^C-DED space, and masked using the individual binary grey matter mask. Registered ^18^F-FDG images in each subject were sampled using the created individual cortical atlas using the whole pons as reference. For ^11^C-DED PET quantification, a modified reference Patlak model [21, 22] was applied to the 20–60 min dynamic ^11^C-DED PET images using the cerebellar grey matter as the modified reference region [23] to generate individual parametric Patlak slope images (units per minute). The model assumed a cerebellar grey matter slope value of 0.01 min^−1^. ^11^C-DED binding was then expressed as the ratio of the ^11^C-DED slope value in the target region of interest to that in the cerebellar grey matter, as previously described [16].

### Statistical analysis

LMEMs were applied to investigate the longitudinal associations between regional ^11^C-DED binding and ^18^F-FDG uptake in 12 bilateral regions of interest in mutation carriers and noncarriers separately. LMEMs were used since models of this type are suitable for longitudinal designs and can flexibly handle samples with missing follow-up data in some subjects. LMEMs were applied separately in mutation carriers and noncarriers, using the model expression:

1 $$ {}^{18}\mathrm{F}-{\mathrm{FDG}}_{\mathrm{ROI}}\sim {\upbeta}_0+{\upbeta_1}^{11}\mathrm{C}-{\mathrm{DED}}_{\mathrm{ROI}}+\mathrm{Random}\ \mathrm{intercept}\;\left(\mathrm{I}\right)+\varepsilon $$

where *β*_0_ and *β*_1_ are fixed-effects coefficients, Random intercept is a variable that takes into account the repeated measures in the same individual subject number *I*, and *ε* is an error term. To further explore whether the regional associations between ^11^C-DED binding and ^18^F-FDG uptake were significantly different between mutation carriers and noncarriers, an interaction analysis was performed using the whole sample of mutation carriers and noncarriers, using the LMEM expression:

2 $$ {\displaystyle \begin{array}{c}{}^{18}\mathrm{F}-{\mathrm{FDG}}_{\mathrm{ROI}}\sim {\upbeta}_0+{\upbeta_1}^{11}\mathrm{C}-{\mathrm{DED}}_{\mathrm{ROI}}+{\upbeta}_2\;\mathrm{Mutation}\ \mathrm{status}+\\ {}+{\upbeta_3}^{11}\mathrm{C}-{\mathrm{DED}}_{\mathrm{ROI}}:\mathrm{Mutation}\ \mathrm{status}\;\left(\mathrm{interaction}\right)+\mathrm{Random}\ \mathrm{intercept}\;\left(\mathrm{I}\right)+\varepsilon \end{array}} $$

where Mutation status is a categorical variable (carrier/noncarrier), *β*_0_, *β*_1_, *β*_2_ and *β*_3_ are fixed-effects coefficients with *β*_3_ representing the coefficient of the interaction term, Random intercept takes into account the repeated measures in the same individual subject number *I*, and *ε* is an error term. In all LMEMs (Eqs. 1 and 2), the covariance matrix of the residuals was modelled by an unstructured covariance matrix, and models were implemented using restricted maximum likelihood estimation with the threshold for statistical significance set at *P* < 0.05. LMEMs were implemented using lme4 version 1.1 and lmerTest version 2.0 packages in R statistical software.

In each LMEM, the conditional coefficient of determination (*R*^2^_c_) was obtained to estimate the variance explained by both fixed and random effects combined. The *R*^2^_c_ values were obtained using the R package MuMIn version 1.4, developed based on a previously published method [24]. Graphical representations of the LMEM results were produced using the ggplot2 package version 2.2.1. All statistical analyses were performed in R, version 3.3.3.

Since the LMEMs were repeated for 12 bilateral regions of interest, the statistical results were further corrected for multiple comparisons using a false discovery rate (FDR) procedure as implemented in the method of Benjamini and Hochberg [25] using pplot software [26].

## Results

**Fig. 1:**
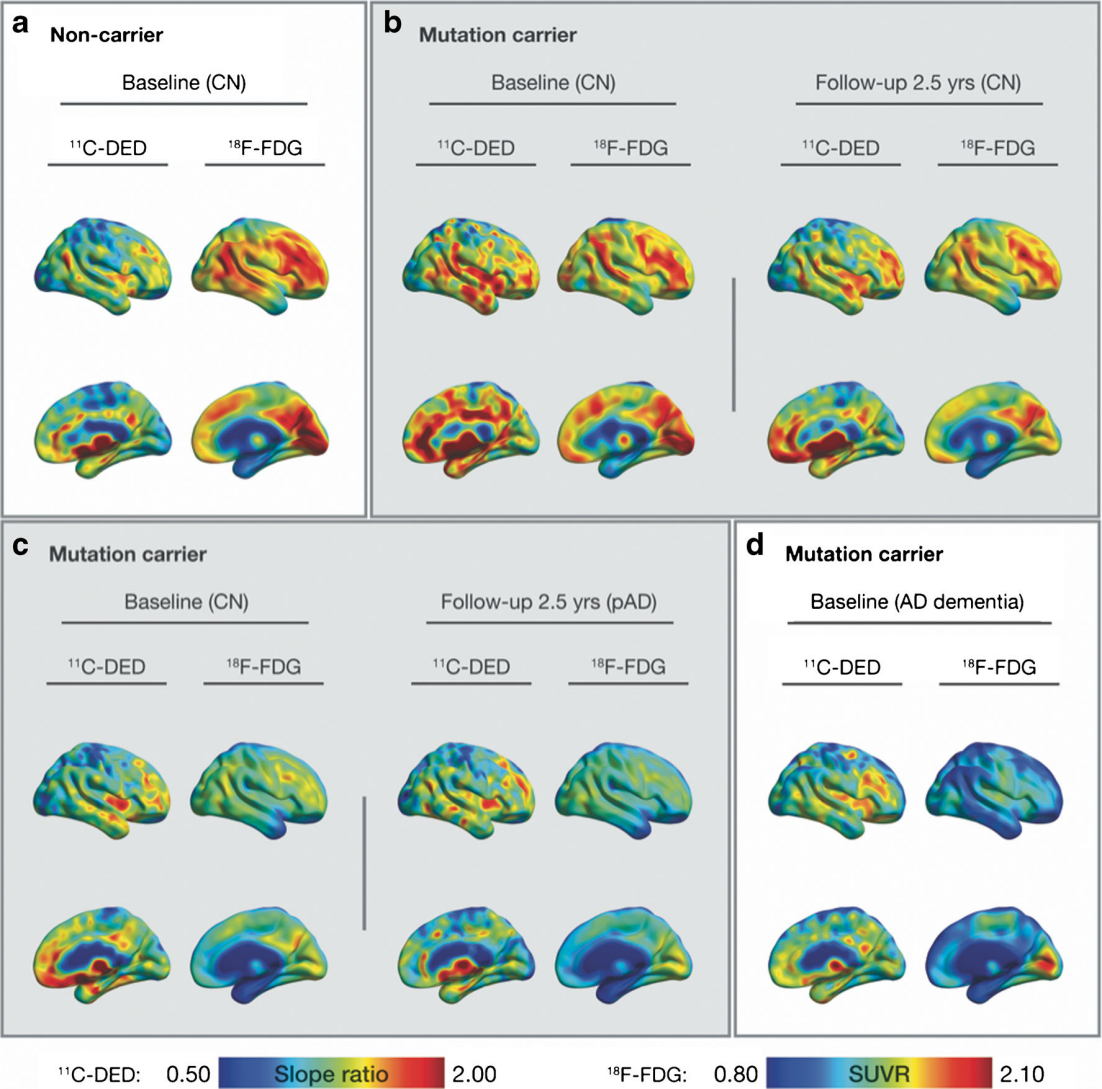
Representative ^11^C-DED and ^18^F-FDG PET images. **a** A noncarrier. **b** A mutation carrier who was asymptomatic both at baseline (around two decades before symptom onset) and at the 2.5-year follow-up. **c** A mutation carrier who was asymptomatic at baseline and who had converted to prodromal AD at the 2.5-year follow-up. **d** A mutation carrier who was symptomatic with a diagnosis of AD dementia about 6 years after onset. *AD* Alzheimer’s disease, *CN* cognitively normal, ^*11*^*C-DED* ^11^C-deuterium-l-deprenyl, ^*18*^*F-FDG* ^18^F-fluorodeoxyglucose, *pAD* prodromal Alzheimer’s disease, *SUVR* standardised uptake value ratio

 Figure 1 shows representative ^11^C-DED and ^18^F-FDG PET scans in one noncarrier (Fig. 1a), two mutation carriers (both asymptomatic at baseline and followed-up longitudinally; Fig. 1b, c), and one symptomatic carrier (Fig. 1d). The PET scans in the noncarrier (Fig. 1a) show metabolically normal ^18^F-FDG uptake and control levels of ^11^C-DED. In mutation carriers, the highest brain levels of ^11^C-DED and ^18^F-FDG were seen in an early presymptomatic subject at baseline, around two decades before onset (Fig. 1b). The uptake of both tracers declined longitudinally in most brain regions, especially in the cingulate cortex and precuneus (Fig. 1b). The second mutation carrier (Fig. 1c) was still asymptomatic about 2 years after the family-specific average age of onset, and showed lower global levels of ^11^C-DED and ^18^F-FDG than the other mutation carrier (Fig. 1b). The uptake of both tracers declined as the mutation carrier converted from asymptomatic to prodromal AD, especially in the frontal and cingulate cortices, and in the precuneus (Fig. 1c). The symptomatic mutation carrier diagnosed with dementia (Fig. 1d) showed low ^18^F-FDG uptake and control levels of ^11^C-DED binding. In noncarriers levels of ^18^F-FDG and ^11^C-DED showed no change visually at any longitudinal follow-up (not shown).

**Fig. 2:**
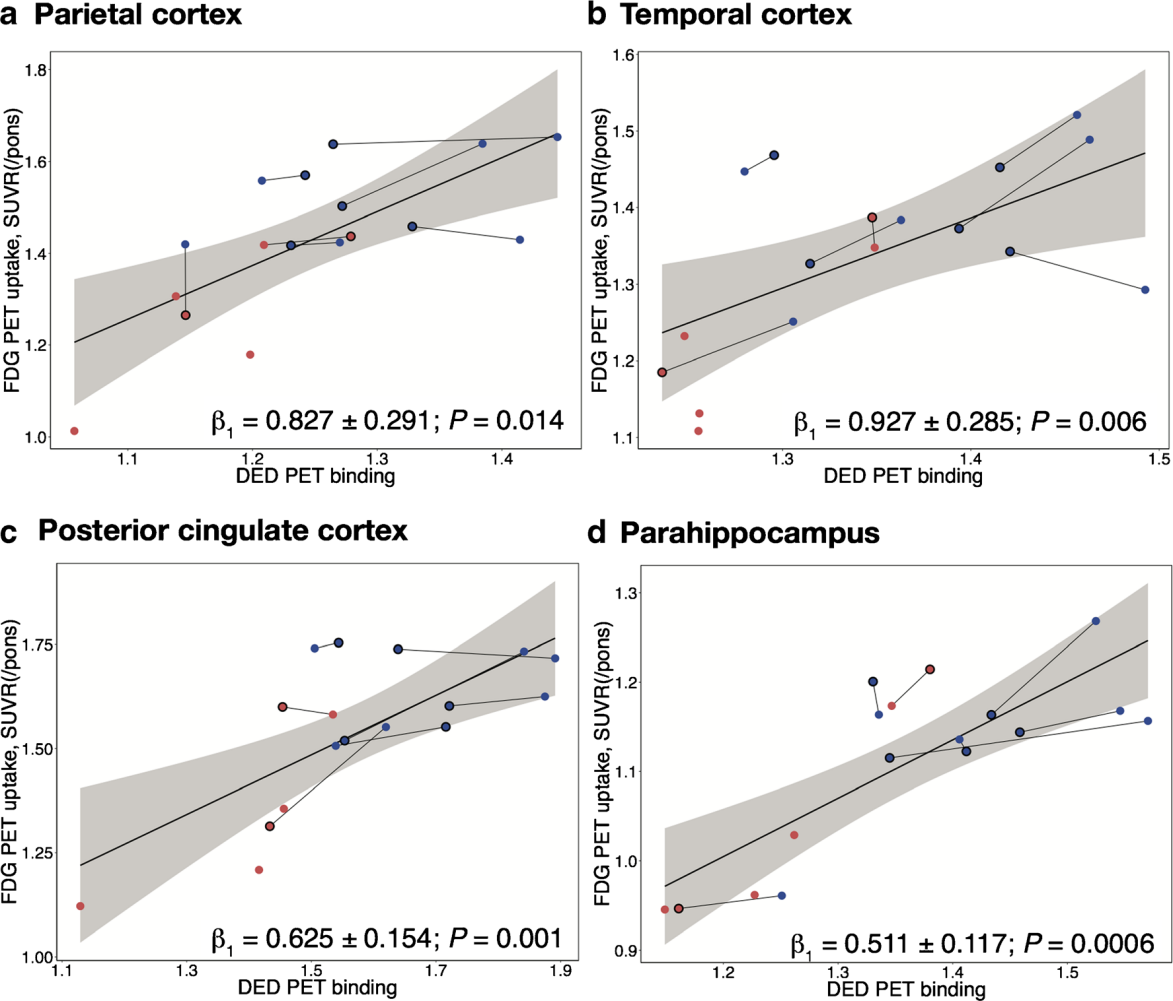
Significant associations between longitudinal ^11^C-DED binding and ^18^F-FDG uptake in mutation carriers. Longitudinal associations in **a** the parietal cortex, **b** the temporal cortex, **c** the posterior cingulate cortex, and **d** the parahippocampus are shown. *Blue circles* presymptomatic mutation carriers, *red circles* symptomatic mutation carriers, *symbols with black outline* follow-up data, *symbols with no outline* baseline data. *DED* ^11^C-deuterium-l-deprenyl, *FDG* ^18^F-fluorodeoxyglucose, *SE* standard error, *SUVR* standardised uptake value ratio

Table 1 shows the LMEM statistical results in mutation carriers. Statistically significant positive associations were found between ^11^C-DED binding and ^18^F-FDG uptake, which remained significant after FDR correction in all regions of interest except the hippocampus and frontal cortex (Table 1). In the frontal cortex the association was significant at the level of a trend (*P* = 0.062). In mutation carriers, the longitudinal decline in ^11^C-DED binding explained a large fraction of the variance in ^18^F-FDG uptake (*R*^2^_c_ ranging from 0.79 to 0.98). Figure 2 illustrates the significant longitudinal associations between ^11^C-DED binding and ^18^F-FDG uptake in four representative regions of interest in mutation carriers. As shown in Supplementary Table 1 and Supplementary Fig. 1, the longitudinal relationships between ^11^C-DED binding and ^18^F-FDG uptake were not significant in mutation noncarriers. The results of the interaction analysis (Eq. 2; Supplementary Table 2) further showed that the regional associations between ^11^C-DED binding and ^18^F-FDG uptake were significantly different between mutation carriers and noncarriers in five regions of interest (temporal, anterior and posterior cingulate cortices, insula and parahippocampus). The results remained significant after FDR correction for multiple comparisons in the temporal, posterior cingulate and parahippocampal cortices.

**Table 1:** Longitudinal associations between ^11^C-DED binding and ^18^F-FDG PET uptake in 12 regions of interest in mutation carriers

Region	Fixed-effects coefficient *β*_1_ (± SE)	*P* value	Degrees of freedom	*t* value	*F*	*R* ^2^_c_
Frontal cortex	0.928 ± 0.456	0.062	13.55	2.03	4.14	0.86
Parietal cortex	**0.827 ± 0.291**	**0.014**	**12.64**	**2.84**	**8.05**	**0.83**
Temporal cortex	**0.927 ± 0.285**	**0.006**	**14.50**	**3.26**	**10.61**	**0.91**
Occipital cortex	**0.863 ± 0.319**	**0.019**	**12.40**	**2.71**	**7.34**	**0.81**
Anterior cingulate cortex	**0.658 + 0.199**	**0.005**	**14.94**	**3.31**	**10.97**	**0.90**
Posterior cingulate cortex	**0.625 ± 0.154**	**0.001**	**13.18**	**4.07**	**16.60**	**0.86**
Insular cortex	**0.392 ± 0.111**	**0.006**	**9.69**	**3.52**	**12.39**	**0.92**
Parahippocampus	**0.511 ± 0.117**	**0.0006**	**14.02**	**4.38**	**19.21**	**0.89**
Caudate nucleus	**0.465 ± 0.119**	**0.001**	**14.56**	**3.92**	**15.37**	**0.86**
Putamen	**0.568 ± 0.093**	**0.0001**	**9.40**	**6.11**	**37.38**	**0.98**
Thalamus	**0.391 ± 0.089**	**0.001**	**12.90**	**4.40**	**19.36**	**0.79**
Hippocampus	0.214 ± 0.161	0.215	9.24	1.33	1.77	0.86

Linear mixed-effects models (LMEMs) were used to assess the longitudinal associations between ^11^C-DED binding and ^18^F-FDG uptake in 12 regions of interest in mutation carriers using the equation: ^18^F-FDG_ROI_ ~ *β*_0_ + *β*_1_^11^C-DED_ROI_ + Random intercept (*I*) + ε*,* where *β*_0_ and *β*_1_ are fixed-effects coefficients, Random intercept is a variable that takes into account the repeated measures in the same individual subject number *I*, and *ε* is an error term.

Associations that were significant after correction for multiple comparisons using the false discovery rate (FDR) are indicated in bold

^*11*^*C-DED* ^11^C-deuterium-l-deprenyl, ^*18*^*F-FDG* ^18^F-fluorodeoxyglucose, *R*^*2*^_*c*_ conditional coefficient of determination, *SE* standard error

## Discussion

The combined evidence from the studies reviewed above and the new analysis reported here suggest that astrocytes potentially contribute significantly to the in vivo ^18^F-FDG PET signal. The evidence indicates a significant association between the processes of decline in astrocyte function, as measured by a reduction in MAO-B, and a progressive reduction in glucose metabolism during the ADAD disease course. Therefore, the observed decline in the functional astrocyte marker MAO-B might indeed reflect reduced glucose demand by astrocytes due to astrodegeneration, an astrocytic phenotype commonly associated with progression of neurodegenerative diseases, and therefore reduced glucose utilization or lactate availability for the adjacent neurons [27]. While the astrocyte marker used in the current study (^11^C-DED) is not a perfect marker for astrocyte function per se, as it measures MAO-B expression and not astrocytic glucose or glutamate uptake, it is the only in vivo marker currently available that is used in dementia research to interrogate the energetic coupling of astrocytes and neurons.

Astrocytes are known to play an important role in the clearance of amyloid-β, as demonstrated in transgenic animal models of AD and in in vitro studies [28, 29]. ADAD is characterised by the early overproduction of amyloid-β species, including soluble amyloid-β oligomers. Interestingly, exposure of astrocytes to soluble amyloid-β_25–35_ (a peptide used in in vitro studies to mimic naturally occurring amyloid-β_1–42_) has been found to lead to increased glucose uptake via glycolysis coupled with lactate release [28], suggesting that the internalisation of amyloid-β peptides by astrocytes significantly alters their metabolic phenotype, with possible deleterious consequences for neuronal function. Exposure of astrocytes to soluble amyloid-β_25–35_ also leads to increased astrocytic MAO-B expression [30]. In a transgenic mouse model of AD, astrocytic MAO-B overexpression resulted in excess GABA (gamma-aminobutyric acid) and excitotoxic glutamate release, disrupting oxidative homeostasis and leading to cognitive deficits [31].

Based on the preclinical evidence reviewed above, it is reasonable to suggest that amyloid-β oligomers in presymptomatic ADAD stimulate MAO-B overexpression in astrocytes, together with the secretion of neurotoxic glutamate and increased glycolysis that contribute to the ^18^F-FDG signal. Interestingly, other in vivo PET studies have shown a similar correspondence between ^11^C-DED binding and ^18^F-FDG uptake, but in separate studies in which the tracers were used in different cohorts. In one study in patients with amyotrophic lateral sclerosis, increased MAO-B expression was observed in the pons and white matter [21]. Hypermetabolism was found in a different cohort of patients with amyotrophic lateral sclerosis involving the pons and midbrain, and was interpreted as probably due to neuroinflammation [32]. In our study, we also observed a late-phase decline in MAO-B expression that was probably a downstream effect of the early MAO-B upregulation and a reflection of chronic neuroinflammation due to excess glutamate, leading to astrocytic dysfunction and atrophy [27]. The lack of significant longitudinal associations between ^11^C-DED binding and ^18^F-FDG uptake in noncarriers found in this study was plausibly due to the lack of variation in ^11^C-DED levels and minimal longitudinal changes in ^18^F-FDG uptake, partly due to the relatively young age of ADAD individuals.

^11^C-DED binding is a reliable marker of astrocytic function in neocortical areas. However, the binding of the tracer in subcortical nuclei (especially the striatum and thalamus) as well as specific subregions of the hippocampus (i.e. the hippocampal uncus) may partially result from the increased concentration of MAO-B in serotonergic cells and may not be just a measure of astrocytosis, and thus the longitudinal associations between ^11^C-DED binding and ^18^F-FDG uptake in mutation carriers should be interpreted with some caution in these regions [33]. However, the observation of positive associations between ^11^C-DED binding and ^18^F-FDG uptake across most brain regions adds to the evidence that astrocytes contribute significantly to the concurrent longitudinal changes in ^11^C-DED binding and ^18^F-FDG uptake across the brain.

The results of previous in vivo and in vitro studies using alternative astrocyte markers are consistent with our findings. For example, levels of the astrocyte-specific glutamate transporters GLT1 (in rodents) and EAAT2 (equivalent to GLT1 in humans) have been reported to be reduced in post-mortem tissue [34–36], indicating loss of function of astrocytes during late disease stages. Similarly, levels of glutamine synthetase (specific to astrocytes) have been found to decline with age in a transgenic mouse model of AD, suggesting impairment in glutamate homeostasis with disease progression [37].

Finally, previous in vivo PET studies add to the evidence for a connection between astrocyte dysfunction and hypometabolism. Patients with glucose transporter deficiency syndrome, a genetic disease characterised by a decrease in expression of GLUT1 (glucose transporter 1) protein (a glucose transporter predominantly expressed in astrocytes), have shown global metabolic decline as measured by ^18^F-FDG PET, suggesting that astrocyte dysfunction is significantly related to the low ^18^F-FDG PET signal [38]. In vivo astrocyte dysfunction measured as reduced aerobic glycolysis has also been observed by PET imaging in patients with Huntington’s disease, and in individuals with preclinical AD together with accumulation of neurofibrillary tau tangles [9], adding to the evidence that astrocyte dysfunction plays an important role in the progression of neurodegenerative diseases.

Caution is obviously required not to overinterpret the data reported here. The two primary weaknesses of the current investigation are the limited sample size of 11 ADAD mutation carriers and, as alluded to above, the fact that ^11^C-DED binding is a measure of one aspect of astrocyte function, namely the overexpression of MAO-B. In spite of the limited sample size, the longitudinal analysis using LMEMs allowed adjustment for between-individual differences and thus reduced this source of variability that is often observed with cross-sectional designs. The LMEM results reveal strong and significant effects in multiple brain regions also after correction for multiple comparisons. However, it is still crucial that these findings are replicated in a larger sample of patients with either AD or another disease affecting astrocytes, and with a PET tracer that may target astrocytic glucose or glutamate uptake.

Presently there are several PET tracers available for imaging microglia, with varying degrees of success, but ^11^C-DED is the only tracer to date considered to measure astrocyte function that has published longitudinal data available [16]. Another promising astrocyte PET biomarker in development is ^11^C-BU99008 [39]. This tracer labels imidazoline receptors predominantly found in astrocytes. In addition to astrocyte markers, PET tracers for investigating synaptic density have been developed. These tracers may be better markers of neuronal integrity than ^18^F-FDG, and include ^11^C-UCB-J, which measures the SV2A (synaptic vesicle glycoprotein 2A) receptor at synapses [40, 41]. These developments are exciting and will very likely provide new insights into dementing diseases such as AD, but they will not allow conclusive testing of the hypothesis that the ^18^F-FDG signal is predominantly driven by astrocyte energy demand. To test this, PET tracers that specifically target the astrocyte-specific glutamate transporters including GLT1/EAAT2 and GLAST (glutamate aspartate transporter) will need to be developed and until such time many questions will remain, including: How early in the neurodegenerative cascade does astrocyte dysfunction occur? Does it precede neuronal dysfunction and is it causal? Answering these questions will go some way to elucidating what ^18^F-FDG PET is actually measuring.

The mounting evidence that astrocytes contribute to the in vivo ^18^F-FDG PET signal and therefore brain glucose metabolism has important clinical implications. New therapies may be targeted at specifically preserving the metabolic function of astrocytes. This suggestion is clearly a tentative one and the authors hope the current work will stimulate further discussion and lead to more research involving comprehensive translational studies in humans using multimodal PET imaging of potential new, specific astrocyte biomarker targets taken from in vitro biochemical studies. Only such comprehensive translational studies will help elucidate the cellular basis of cerebral glucose metabolism.

## Electronic supplementary material

ESM 1 (PDF 331 kb)

## Abbreviations

AD: Alzheimer’s disease
ADAD: Autosomal dominant Alzheimer’s disease
^11^C-DED: ^11^C-Deuterium-l-deprenyl
EYO: Estimated years to symptom onset
^18^F-FDG: ^18^F-Fluorodeoxyglucose
LMEM: Linear mixed-effects model
MAO-B: Monoamine oxidase B
^11^C-PiB: ^11^C-Pittsburgh compound-B
FDR: False discovery rate
