# Supplementary material for: Longitudinal association between astrocyte function and glucose metabolism in autosomal dominant Alzheimer’s disease
Source: Eur J Nucl Med Mol Imaging. 2018 Dec 4;46(2):348–56. doi: 10.1007/s00259-018-4217-7 (PMC6333721; doi:10.1007/s00259-018-4217-7)
Supplement: Supplementary file 1 — (PDF 331 kb) [file 259_2018_4217_MOESM1_ESM.pdf]

Supplementary Material for

Longitudinal association between astrocyte function and glucose metabolism in autosomal dominant Alzheimer's disease

European Journal of Nuclear Medicine and Molecular Imaging

Authors: Stephen F. Carter[1], Konstantinos Chiotis[2,3], Agneta Nordberg[2,4], Elena Rodriguez-Vieitez[2]

[1] Wolfson Molecular Imaging Centre, Division of Neuroscience and Experimental Psychology, University of Manchester, M20 3LJ Manchester, United Kingdom

[2] Karolinska Institutet, Department of Neurobiology, Care Sciences and Society, Division of Clinical Geriatrics, 141 52 Stockholm, Sweden

[3] Theme Neurology, Karolinska University Hospital, 171 76 Stockholm, Sweden

[4] Theme Aging, Karolinska University Hospital, 141 86 Stockholm, Sweden

Corresponding author: Elena Rodriguez-Vieitez, elena.rodriguez-vieitez@ki.se

Supplementary Table 1 Longitudinal relationships between ^{11}C-DED binding and ^{18}F-FDG uptake in 12 regions of interest in mutation noncarriers

Region	Fixed-effects coefficient, β_1 (± SE)	*P* value	Degrees of freedom	*t* value	*F*	R^2_c
Frontal cortex	0.244 ± 0.270	0.381	15.07	0.90	0.82	0.73
Parietal cortex	0.183 ± 0.233	0.442	16.76	0.79	0.62	0.65
Temporal cortex	-0.016 ± 0.193	0.934	14.83	-0.08	0.01	0.70
Occipital cortex	0.387 ± 0.223	0.099	17.91	1.74	3.02	0.73
Anterior cingulate cortex	0.042 ± 0.193	0.829	16.88	0.22	0.05	0.74
Posterior cingulate cortex	0.009 ± 0.176	0.961	16.95	0.05	0.002	0.60
Insular cortex	0.042 ± 0.138	0.764	17.96	0.31	0.09	0.77
Parahippocampus	0.021 ± 0.081	0.801	16.01	0.26	0.07	0.70
Caudate nucleus	0.287 ± 0.137	0.057	12.74	2.09	4.37	0.91
Putamen	0.107 ± 0.115	0.367	16.65	0.93	0.86	0.58
Thalamus	0.132 ± 0.123	0.301	15.83	1.07	1.14	0.59
Hippocampus	-0.005 ± 0.062	0.939	15.13	-0.08	0.01	0.55

Linear mixed-effects models (LMEMs) were used to assess the longitudinal relationships between ^{11}C-DED binding and ^{18}F-FDG uptake in 12 regions of interest in mutation noncarriers using the equation: $^{18}\text{F-FDG}_{\text{ROI}} \sim \beta_0 + \beta_1\ ^{11}\text{C-DED}_{\text{ROI}}$ + Random intercept (I) + ε, where β_0 and β_1 are fixed-effects coefficients, Random intercept is a variable that takes into account the repeated measures in the same individual subject number I, and ε is an error term.

^{11}C-DED = ^{11}C-deuterium-L-deprenyl; ^{18}F-FDG = ^{18}F-fluorodeoxyglucose; R^2_c = conditional coefficient of determination; SE = standard error

Supplementary Table 2 Results of the linear mixed-effects models to investigate the interaction of Mutation status (carrier/non-carrier) on the longitudinal relationships between ^{11}C-DED binding and ^{18}F-FDG uptake in 12 regions of interest in the whole sample of mutation carriers and noncarriers

**Region**	**Fixed-effects coefficient of interaction term, β_3 (± SE)**	***P* value**	**Degrees of freedom**	***t* value**	***F***	**R^2_c**
Frontal cortex	0.746 ± 0.514	0.158	28.86	1.45	2.11	0.83
Parietal cortex	0.754 ± 0.373	0.052	32.87	2.02	4.08	0.80
Temporal cortex	**0.986 ± 0.351**	**0.008**	**32.22**	**2.81**	**7.91**	**0.87**
Occipital cortex	0.485 ± 0.385	0.218	29.97	1.26	1.59	0.81
Anterior cingulate cortex	0.616 ± 0.282	0.036	32.66	2.18	4.76	0.85
Posterior cingulate cortex	**0.698 ± 0.238**	**0.006**	**32.99**	**2.93**	**8.59**	**0.83**
Insular cortex	0.411 ± 0.188	0.037	27.80	2.19	4.79	0.88
Parahippocampus	**0.515 ± 0.150**	**0.002**	**32.87**	**3.43**	**11.80**	**0.84**
Caudate nucleus	0.140 ± 0.190	0.466	30.09	0.74	0.54	0.89
Putamen	0.444 ± 0.241	0.075	32.77	1.84	3.39	0.77
Thalamus	0.260 ± 0.159	0.112	29.27	1.64	10.78	0.71
Hippocampus	0.231 ± 0.176	0.201	28.58	1.31	1.71	0.79

Linear mixed-effects models (LMEMs) were used to assess the interaction of Mutation status (carrier/non-carrier) on the longitudinal relationship between ^{11}C-DED binding and ^{18}F-FDG uptake in 12 regions of interest, using the equation: $^{18}\text{F-FDG}_{ROI} \sim \beta_0 + \beta_1\ {}^{11}\text{C-DED}_{ROI} + \beta_2$ Mutation status + $\beta_3\ {}^{11}\text{C-DED}_{ROI}$: Mutation status (interaction) + Random intercept (I) + ε, where Mutation status is a categorical variable (carrier/non-carrier), β_1, β_2 and β_3 are fixed-effects coefficients with β_3 representing the coefficient of the interaction term, Random intercept takes into account the repeated measures in the same individual subject number I, and ε is an error term. Associations that were significant after multiple comparisons correction using false discovery rate (FDR) are indicated in bold.

^{11}C-DED = ^{11}C-deuterium-L-deprenyl; ^{18}F-FDG = ^{18}F-fluorodeoxyglucose; R^2_c = conditional coefficient of determination; SE = standard error

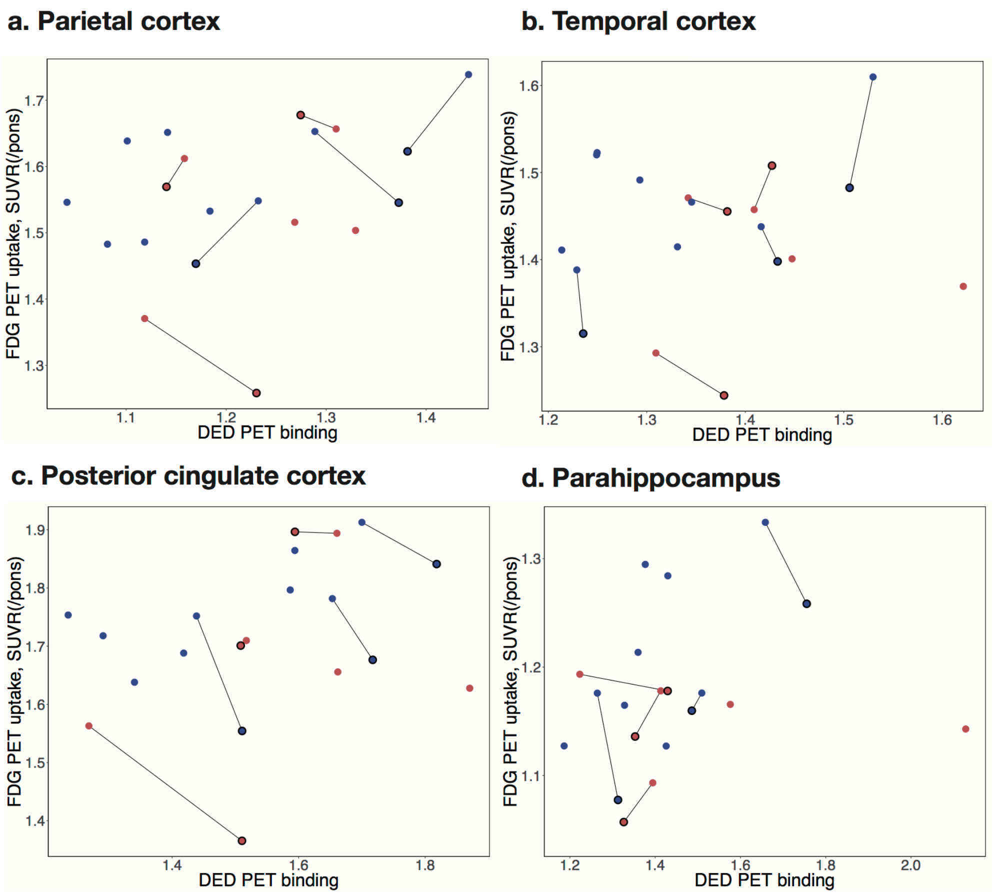

Supplementary Fig. 1 Scatterplots illustrating the longitudinal patterns of ^{11}C-DED binding and ^{18}F-FDG uptake in mutation noncarriers. The longitudinal relationships are illustrated in the following regions: **(a)** parietal cortex, **(b)** temporal cortex, **(c)** posterior cingulate cortex, and **(d)** parahippocampus. Blue circles = presymptomatic mutation carriers; red circles = symptomatic mutation carriers; symbols with black outline = follow-up data; symbols with no outline = baseline data.

DED = ^{11}C-deuterium-L-deprenyl; FDG = ^{18}F-fluorodeoxyglucose; SE = standard error; SUVR = standardised uptake value ratio
